# The Development of High-Density Vertical Silicon Nanowires and Their Application in a Heterojunction Diode

**DOI:** 10.3390/ma9070534

**Published:** 2016-06-30

**Authors:** Wen-Chung Chang, Sheng-Chien Su, Chia-Ching Wu

**Affiliations:** 1 Department of Electronic Engineering, Southern Taiwan University of Science and Technology, Tainan 71005, Taiwan; changwc@stust.edu.tw (W.-C.C.); ct6288@gmail.com (S.-C.S.); 2 Department of Electronic Engineering, Kao Yuan University, Kaohsiung 82151, Taiwan

**Keywords:** silicon nanowire, indium tin oxide, indium zinc oxide, heterojunction diode

## Abstract

Vertically aligned p-type silicon nanowire (SiNW) arrays were fabricated through metal-assisted chemical etching (MACE) of Si wafers. An indium tin oxide/indium zinc oxide/silicon nanowire (ITO/IZO/SiNW) heterojunction diode was formed by depositing ITO and IZO thin films on the vertically aligned SiNW arrays. The structural and electrical properties of the resulting ITO/IZO/SiNW heterojunction diode were characterized by field emission scanning electron microscopy (FE-SEM), X-ray diffraction (XRD), and current−voltage (I−V) measurements. Nonlinear and rectifying I−V properties confirmed that a heterojunction diode was successfully formed in the ITO/IZO/SiNW structure. The diode had a well-defined rectifying behavior, with a rectification ratio of 550.7 at 3 V and a turn-on voltage of 2.53 V under dark conditions.

## 1. Introduction

Nanostructured solar cells (containing nanospheres [1], nanowires [2,3,4], or nanopillars [5]) have recently been proposed as promising candidates for solar energy harvesting. Silicon (Si) is still the leading material in today’s photovoltaic industry. As the process has matured, silicon nanowire (SiNW) or silicon nanorod (SiNR) structures have become the focus of nanowire solar cells. Si is by far the most versatile and widely used semiconductor—despite the development of many compound semiconductors—due to its distinct advantages, such as abundance, stability, and ease of processing [6,7,8]. Uniform, vertically aligned SiNWs are promising building blocks for a range of vertical devices, including surround-gate field-effect transistors [9], solar cells [10], and thermoelectric modules [11]. Nanostructured solar cells made using low-cost materials are expected to be used in the industry. Polycrystalline nanowire-array solar cells are expected to enhance solar cell efficiency despite their very low material cost, due to their enlarged p–n junction area and suppressed light reflection. Vertically aligned SiNWs can be fabricated with a relatively high degree of control and uniformity through both top-down etching and bottom-up epitaxial growth methods [12]. However, SiNW solar cells have major drawbacks: their carrier collection efficiency is low, and fabricated nanowire cannot be easily coated with a transparent electrode.

Zinc oxide (ZnO) is an n-type semiconductor with a large binding energy of 60 meV and a wide bandgap of 3.3 eV in the UV range. ZnO has numerous applications in optoelectronic devices, including ultraviolet (UV) visible photodetectors [13,14], solar cells [15], light-emitting diodes (LEDs) [16], and flat-panel displays [17]. Fabricating p-type ZnO is difficult, due to the low solubility of the dopants. Most ZnO-based optoelectronic devices rely on heterojunctions between n-type ZnO and p-type semiconducting materials, the most common choice being p-type silicon. Heterojunction n-ZnO/p-Si devices have been employed as UV visible photodetectors [18], solar cells [19,20,21], and LEDs [22].

In this work, we fabricated silicon nanowires using a top-down method: metal-assisted chemical etching (MACE) [23]. This approach is simple and can produce homogenous silicon nanowires. Silver is the most commonly used metal catalyst. Previously, researchers have reported depositing ZnO on SiNW substrates using various techniques, such as atomic layer deposition (ALD) [11,24,25,26], chemical vapor deposition [27], solution synthesis [28], and radio frequency (RF) sputtering [29,30], to fabricate ZnO/SiNW heterojunction devices. We deposited an IZO thin film on SiNW substrates using RF sputtering to form IZO/SiNW heterojunction diodes. Previous studies have shown that the ITO/p-Si heterojunction device exhibits great photovoltaic effect and rectifying behavior [31]. Therefore, the resulting ITO-coated SiNW-based heterojunction device had a very large surface area and a short carrier collection path that enhanced light trapping and increased carrier collection efficiency. Thus, we achieved a significant enhancement in heterojunction diode properties using ITO/IZO/SiNWs.

## 2. Experimental Procedures

**Figure 1:**
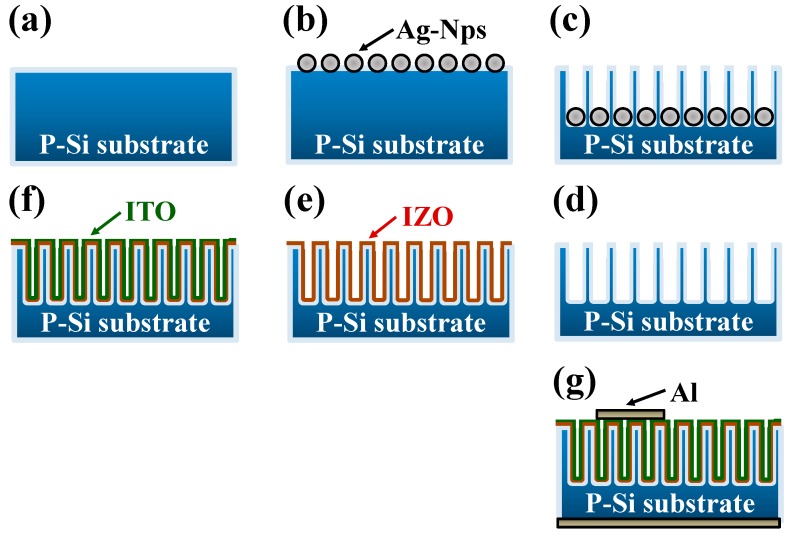
A schematic illustration of the procedure for fabricating the ITO/IZO/silicon nanowire (SiNW) heterojunction diode device.

P-type silicon nanowires (SiNWs) were fabricated through metal-assisted chemical etching (MACE) [23]. Figure 1 shows a schematic illustration of the procedure for fabricating SiNW-based heterojunction devices. Briefly, single crystalline p-Si (100) wafers (2–4 Ω·cm) were cut into rectangular slices of 2 × 2 cm^2^; the slices were subsequently cleaned ultrasonically in acetone, isopropyl alcohol, and deionized water, then dried with nitrogen (N_2_) gas, as shown in Figure 1a. The cleaned silicon slices were immersed in a solution containing hydrogen fluoride (HF) and silver nitrate (AgNO_3_) (HF:AgNO_3_ = 5:0.02 M) to deposit silver (Ag) particles, which acted as the catalyst in the following etching process. Subsequently, (Figure 1b,d), this silicon with Ag particles was etched in an aqueous solution of HF:AgNO_3_ for 10 min to produce a vertical p-Si nanowire. To remove the capped silver, the as-prepared SiNWs were dipped in a nitric acid (HNO_3_) aqueous solution for 90 s. Finally, the SiNWs were rinsed with deionized water and blown dry in N_2_. Figure 1e represents an indium zinc oxide (IZO) thin film being deposited on a SiNW substrate to form a heterojunction diode with a ZnO:In_2_O_2_ = 98:2 mol % ceramic target (Shonan Electron Material Laboratory Corporation, Kanagawa, Japan) using a radio frequency (RF) magnetron sputtering system. The working distance between the SiNW substrate, and the target was fixed at 15 cm. The base pressure was 8 × 10^−^^6^ torr, and the working pressure was 2 × 10^−2^ torr. The deposition temperature of the IZO thin films was room temperature, the RF power was 100 W, and the deposition time was 1 h. The ITO thin films were then deposited on the IZO/SiNW substrates under the same deposition conditions, except that the deposition time was extended to 2 h (Figure 1f). Finally, aluminum (Al) electrodes were deposited on the top and bottom using a thermal evaporation method (Figure 1g). The morphologies of the SiNWs, ITO, IZO, and ITO/IZO/SiNWs were observed using field emission scanning electron microscopy (FESEM, JEOL JSM-6700F, Akishima-shi, Japan). The core/shell nanowire structure of the ITO/IZO/SiNWs was observed using the focused ion beam microscopy (FIB, FEI 650, Hillsboro, OR, USA). The crystalline structures of the ITO and IZO thin films were determined with an X-ray diffractometer (XRD, LabX, Midland, ON, Canada) using CuKα radiation (Kα = 1.5418 Å). Current–voltage (I–V) measurements were performed for the IZO/Si, ITO/IZO/Si, IZO/SiNW and ITO/IZO/SiNW heterojunction structures at room temperature using a Keithley 2400 SourceMeter (Keithley, Beaverton, OR, USA).

## 3. Discussion

**Figure 2:**
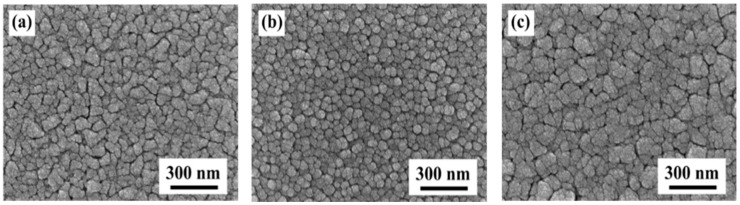
Surface scanning electron microscopy (SEM) images of the thin films: (**a**) ITO/Si; (**b**) IZO/Si; and (**c**) ITO/IZO/Si.

The surface morphologies of the ITO, IZO, and ITO/IZO thin films deposited on the Si substrate are shown in Figure 2. The ITO thin film in Figure 2a shows that the Si substrate was entirely covered with grains of different shapes and sizes, ranging from about 40 to 90 nm. The surface morphology of the IZO film is smooth and compact, with cobble-type grains that have an average grain size of about 52 nm (Figure 2b). However, the ITO/IZO thin film has a larger grain size; abnormal grains were formed and the roughness increased, as shown in Figure 2c.

**Figure 3:**
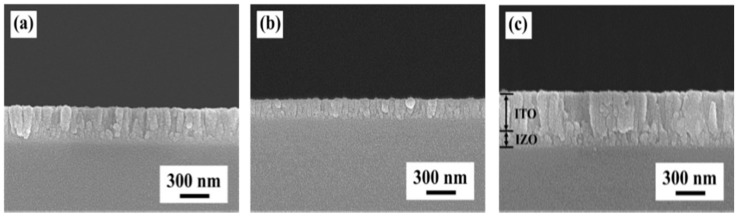
Cross-sectional SEM images of the thin films: (**a**) ITO/Si; (**b**) IZO/Si; and (**c**) ITO/IZO/Si.

Figure 3 presents the sectional morphologies of the ITO, IZO, and ITO/IZO thin films deposited on the Si substrate. Thickness of the ITO thin film was about 250 nm when the deposition time was 1 h Figure 3a. At 2 h, as shown in Figure 3b, thickness of the IZO thin film was about 150 nm. The crystallization in the ITO and IZO thin films displayed preferential orientation growth with a columnar structure. Figure 3c shows that the ITO thin film deposited on the IZO/Si substrate was about 400 nm thick.

**Figure 4:**
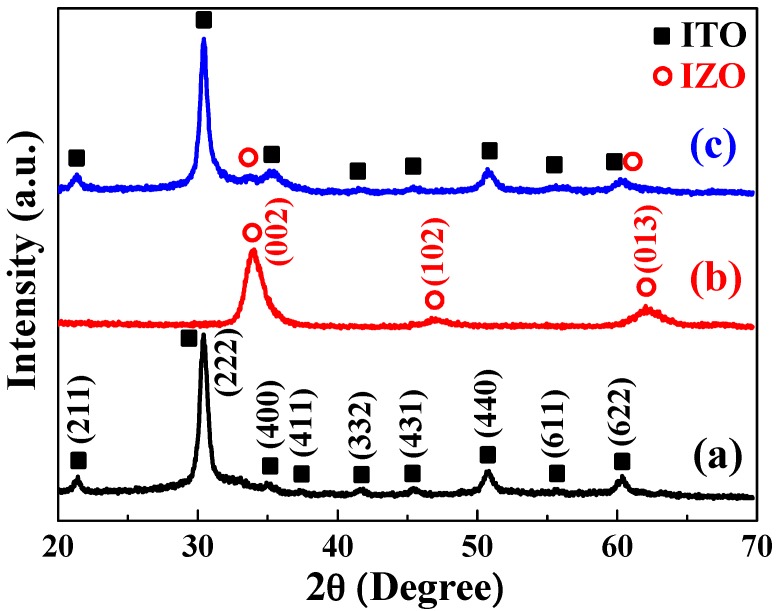
X-ray diffraction (XRD) analyses of thin films: (**a**) ITO; (**b**) IZO; and (**c**) ITO/IZO.

The X-ray diffraction (XRD) patterns of the ITO and IZO films deposited on the Si substrate are presented in Figure 4. The diffraction peaks at 2θ values of 21.4°, 30.3°, 35.1°, 37.2°, 41.7°, 45.4°, 51.6°, 55.6°, and 60.4° in Figure 4a correspond to the (211), (222), (400), (411), (332), (431), (440), (411), and (422) planes of the ITO thin film (JCPDS No. 6-416), respectively. No second phase was present in this film. The IZO film exhibited a dominant (002) peak, with slight (102) and (103) peaks in the diffraction angle (2θ) range of 20°–70° (JCPDS No. S6-314), as shown in Figure 4b. The IZO (002) peak indicated a preferential crystallization orientation with a hexagonal structure along the c-axis at diffraction angles (2θ) near 34.1°; no characteristic peak of the In_2_O_3_ phase was found. As shown in Figure 4c, for the ITO/IZO thin film, ITO diffraction peaks were observed at 21.4°, 30.3°, 35.1°, 37.2°, 41.7°, 45.4°, 51.6°, 55.6°, and 60.4°, along with IZO diffraction peaks at 33.7° and 62.1°.

**Figure 5:**
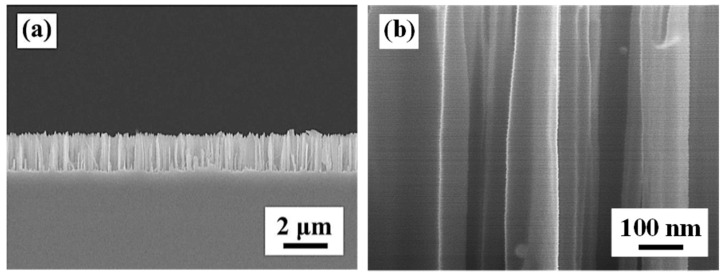
Cross-sectional SEM images of the SiNWs. (**a**) Low rate and (**b**) high rate.

The SEM images in Figure 5 show cross sections of the SiNWs formed after 10 min of etching. The large area of vertically aligned SiNW arrays with uniform length was successfully fabricated through metal-assisted chemical etching (MACE) of Si wafers. Figure 5a indicates that the length of the SiNWs is about 1.7 μm, and the average diameter is about 120 nm (full width at half length of SiNW). In addition, the SiNWs have smooth surfaces with almost no pores as shown in Figure 5b. The result is identical to that produced by Xiaopeng Qi et al. [32].

**Figure 6:**
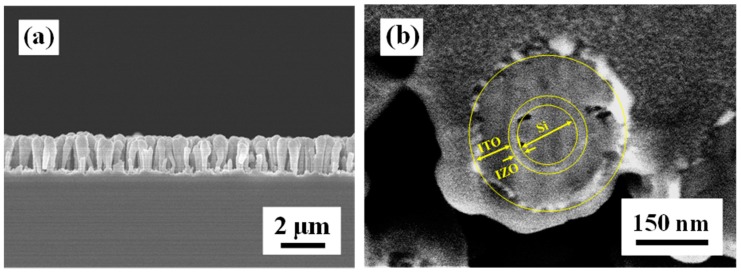
(**a**) Cross-sectional SEM image of the SiNWs and (**b**) the FIB image of the ITO/IZO/SiNWs.

Figure 6a presents a cross-sectional SEM image of the ITO/IZO/SiNW heterojunction structure, showing a “chicken thigh” morphology; because the degree of step coverage in the sputtering method was not good, the ions could not uniformly cover the SiNWs. The focused ion beam (FIB) image of the ITO/IZO/SiNW heterojunction structure is shown in Figure 6b. It can be observed that the diameter of the SiNWs is about 120 nm (full width at half length of SiNW) and the ITO and IZO-coated radial Si NW heterojunction structure.

**Figure 7:**
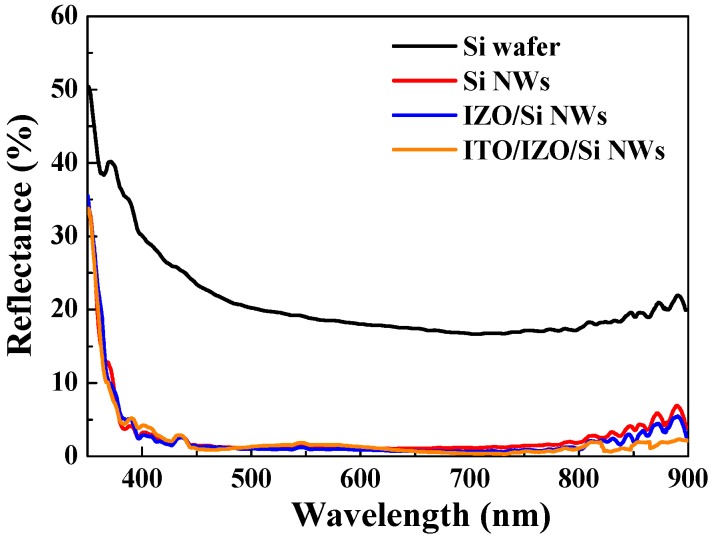
Reflectance of the different structures: Si wafer, SiNWs, IZO/SiNWs, and ITO/IZO/SiNWs.

Figure 7 shows the reflectance of the different Si-base structures. In the visible light range (450–750 nm), compared to the Si wafer (average reflectance about 28%), the SiNW shows a lower average reflectance and the reflectance is about 1.6%. In addition, the average reflectance of the IZO/SiNWs and ITO/IZO/SiNWs are 1.2% and 0.7%, respectively. From the above results, it is demonstrated that the suppressed light reflection is due to the nanowire structure.

**Figure 8:**
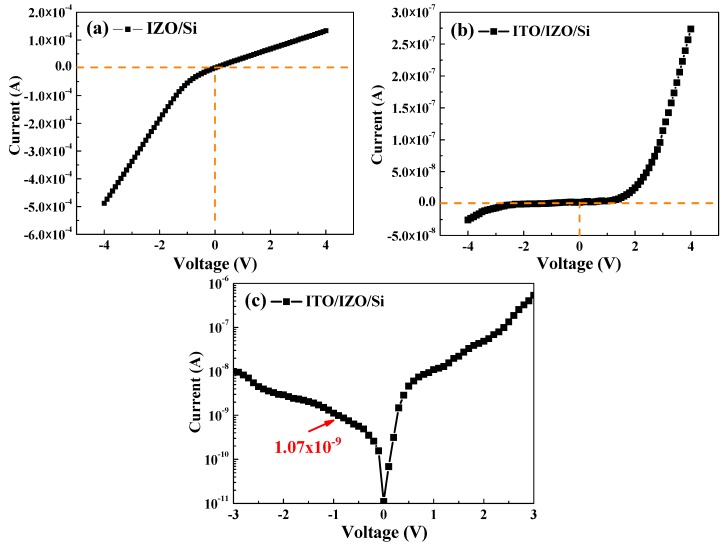
I−V characterization curves of the (**a**) IZO/Si and (**b**) ITO/IZO/Si heterojunction diodes; (**c**) the log(|I|)−V curve of the ITO/IZO/Si heterojunction diode.

Current–voltage (I–V) characterization of the IZO/Si and ITO/IZO/Si was carried out at room temperature, as shown in Figure 8. The nonlinear and rectifying I–V characteristics shown in Figure 8 confirmed that a p–n junction structure had been formed in IZO/Si and ITO/IZO/Si. In Figure 8a, the rectification properties of the IZO/Si heterojunction diode is not obtained. The rectification ratios (R) of the ITO/IZO/Si heterojunction diode (at ±3 V) were calculated using Equation (3), yielding values of 125.6 [33]:

(1) $$R=\frac{\text{Forward current}}{\text{Reverse current}}$$

Figure 8a shows the I−V characteristics of the IZO/Si heterojunction diode. The I−V curve for the turn-on voltages in the forward bias range is indistinct. These indicate that the I−V characteristics of the IZO/Si heterojunction diode did not follow a typical p–n diode curve. Figure 8b shows that under forward bias, the low value of the turn-on voltage for the ITO/IZO/Si heterojunction diode has a low value of about 1.42 V and a higher rectification ratio. The log(|I|)−log(V) curve is plotted in Figure 8c and shows a leakage current of 1.07 × 10^−9^ A/cm^2^ at 1 V for the ITO/IZO/Si heterojunction diode and smaller than the IZO/Si heterojunction diode. The roles of the ITO layer are interpreted with the help of the following key parameters: The electrode area ratio of the ITO/IZO/Si heterojunction diode is much larger than that of the IZO/Si heterojunction diode. As the ITO layer covers the entire surface of the IZO/Si, although the resistivity of ITO film (5 × 10^−^^3^ Ω·cm) is larger than that of Al film (10^−^^5^ Ω·cm), the generated carriers are readily collected via the shortest path between the p–n heterojunction and the ITO layer and effectively transported to the Al finger electrode via ITO without recombination. Therefore, the ITO/IZO/Si heterojunction diode obtained the rectifying I–V curve and small leakage current.

**Figure 9:**
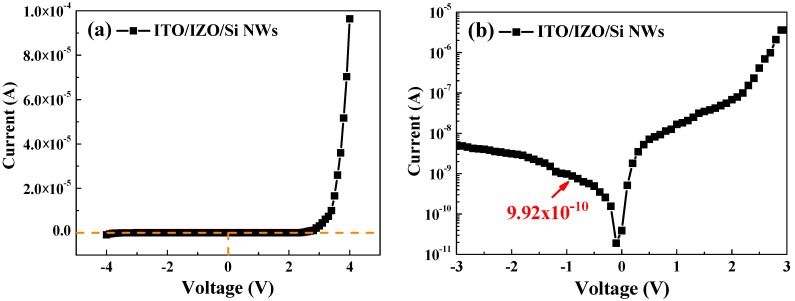
(**a**) I−V characterization curve of ITO/IZO/SiNWs; (**b**) log(|I|)−V curve of ITO/IZO/SiNWs.

**Figure 10:**
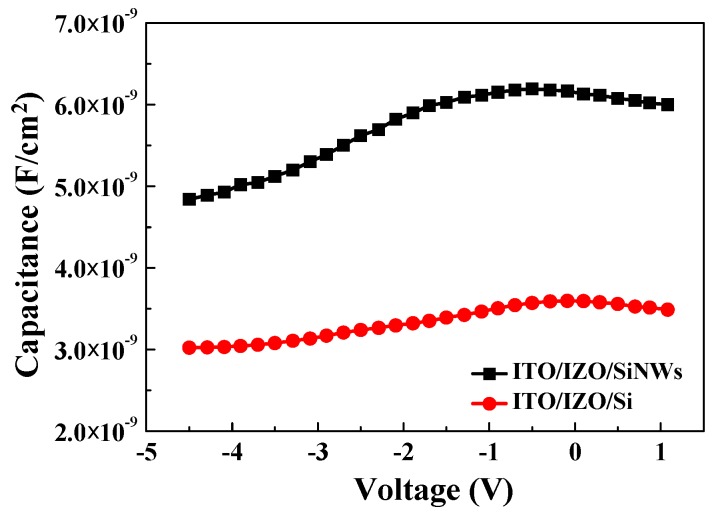
C–V measurement of the ITO/IZO/Si and ITO/IZO/SiNW heterojunction diode.

To improve the properties of the heterojunction diode, SiNWs were used. Figure 9 presents the I–V characteristics of the ITO/IZO/SiNW heterojunction diode. The I–V curve of ITO/IZO/SiNWs clearly shows excellent rectification behavior, with a rectification ratio of about 550.7 at 3 V. The ITO/IZO/SiNW heterojunction diode shows a turn-on voltage of 2.53 V in the forward bias range. When compared with the ITO/IZO/Si heterojunction diode, the ITO/IZO/SiNW heterojunction diode shows a small leakage current in the reverse bias region. This is caused by the junction area of the ITO/IZO/SiNW heterojunction diode being larger than that of the ITO/IZO/Si heterojunction diode. The junction area of the heterojunction diode is characterized using the capacitance–voltage (C−V) method [34,35], as shown in Figure 10. The maximum capacitance (*C*_max_) of the ITO/IZO/SiNW heterojunction diode is about 6.21 nF/cm^2^ and is larger than that of the ITO/IZO/Si heterojunction diode (about 3.58 nF/cm^2^). The ratio of *C*_max_ is proportional to the ratio of the p–n junction areas of the ITO/IZO/SiNWs and the ITO/IZO/Si heterojunction diode [35]. It is understood that the junction area of the ITO/IZO/SiNW heterojunction diode is about 1.73 times that of the ITO/IZO/Si heterojunction diode.

The nonlinear I–V characteristics with diode behavior can be described by a thermionic emission (TE) model theory. The current in such a device can be expressed as [36]

(2) $$I=I_{s}\left[e^{\left(\frac{qV}{nkT}\right)}-1\right]$$

where, *I_s_* is the saturation current; *k* is the Boltzmann constant; *n* is the ideality factor; *T* is the temperature in Kelvin; *q* is the electron charge; and *V* is the applied voltage. The *I_s_* of the heterojunction diode is expressed by the following equation [37]:

(3) $$I_{s}=AA^{*}T^{2}e^{\left(\frac{-q\varphi_{b}}{kT}\right)}$$

where *A* is the device area; A^*^ is the Richardson constant; and $\varphi_{b}$ is the barrier height. The plot of log I vs. V gives the value of the ideality factor. The $\varphi_{b}$ is obtained by rewriting Equation (3) as

(4) $$\varphi_{b}=\frac{kT}{q}ln\left[\frac{AA^{*}T^{2}}{I_{s}}\right]$$

Using Equations (2)–(4), the values of $\varphi_{b}$ and *n* of the ITO/IZO/SiNW heterojunction diode were calculated. The *n* = 1.53 can be calculated from the slope of the linear region of the I–V curve in the forward bias region, and the value of $\varphi_{b}$ is estimated to be 0.91 eV. The high ideality factor could be due to the accelerated recombination of electrons and holes in the depletion region or by the presence of the interfacial layer [38].

## 4. Conclusions

In this study, the rectifying current–voltage (I–V) characteristics confirmed that a p–n junction structure had been formed in ITO/IZO/Si and ITO/IZO/SiNW heterojunction structures. To enhance the properties of the ITO/IZO/Si heterojunction diodes, an array of vertically aligned SiNWs was fabricated through metal-assisted chemical etching. Subsequently, ITO and IZO thin films were de $\varphi_{b}$ posited onto the SiNWs using a radio frequency sputtering technique to create an ITO/IZO/SiNW heterojunction diode. The ITO/IZO/SiNW heterojunction diode showed a lower turn-on voltage (2.53 V) in the forward bias range and a small leakage current in the reverse bias range. Its rectification ratio is 550.7 at 3 V. The ideality factor (*n* = 1.53) can be calculated from the slope of the linear region of the I–V curve in the forward bias region and the barrier height ($\varphi_{b}$) is estimated to be 0.91 eV.
